# Which should be the first-line treatment for *Helicobacter pylori* in Colombia? A lesson from a recent study

**Published:** 2019-12-30

**Authors:** Rinaldo Pellicano

**Affiliations:** Unit of Gastroenterology, Molinette-SGAS Hospitals, Via Cavour 31, 10126 Turin, Turin 10123, Italy Unit of Gastroenterology Molinette-SGAS Hospitals TurinTurin Italy

Turin, August 30th, 2019

Dear Editor,

In the last decades, the recommended regimens for *Helicobacter pylori* eradication have included the combination of a proton-pump inhibitor (PPI) and two or more antibiotics ^1^. Among the latter, clarithromycin is widely used to treat *H. pylori* infection, due to its low minimal inhibitory concentration. However, due to a steady increase in *H. pylori* resistance to clarithromycin, this drug has become progressively less efficacious worldwide.

To highlight the concerns caused by the increasing resistance of this bacterium to antibiotics, the World Health Organization inserted *H. pylori* with high priority for clarithromycin resistance, in the priority list for research and development of new antibiotics ^2^. A European multicentre study, published in 2013, showed that the resistance rate of *H. pylori* in Europe was 34.9% for metronidazole, 17.5% for clarithromycin, 14.1% for levofloxacin, 1.1% for rifabutin, 0.9% for tetracycline, and 0.7% for amoxicillin ^3^.

The dominant mechanisms underlying the development of clarithromycin resistance are several point mutations in domain V of the *23S ribosomal RNA* (*rRNA*) gene, which result in decreased affinity and in absence of clarithromycin binding to the 50s ribosome subunit, and thus, failure to influence protein synthesis.

It is well-known that clarithromycin resistance may originate from the previous consumption of macrolides. There are essential point mutations, which can occur at the nucleotide positions 2142 (A2142G and A2142C), 2143 (A2143G) and 2144 (A2144G) in the peptidyl transferase loop of the *23S rRNA* gene. These mutations result in conformational change leading to decreased efficacy of the drug ^4^.

In a recent interesting article, Roldán, *et al*., reported the frequency of A2143G and A2142G mutations in patients with previous unknown *H. pylori* status, admitted for dyspepsia in an endoscopic unit in Medellín, Colombia. They found a prevalence of 44.2% of *H. pylori* infection with A2143G and A2142G mutations in the 18.8% of them ^5^. These results must be considered together with the data regarding the high rate (78%) of RdxA nitroreductase mutations (associated with metronidazole resistance) shown in *H. pylori* strains in Colombia ^6^.

Considering that beyond its involvement in several gastro-duodenal diseases, *H. pylori* is recognized as a necessary but insufficient cause of gastric cancer, it is possible that eradication at a population level may lead to the future decline of this malignancy, especially in countries where it represents a severe burden. Hence, it is crucial to optimize the treatment in each country.

These findings indicate that also in Colombia should be appropriate to treat patients with new therapeutic options, in particular the formulation with bismuth subcitrate potassium, metronidazole, and tetracycline contained in a single capsule (three-in-one). Due to its efficacy the International Guidelines recommended this regimen as first line and second line therapies in regions where clarithromycin resistance has resulted in low-cure rates ^7^.

Very truly yours,
