# Supplementary material for: Which should be the first-line treatment for Helicobacter pylori in Colombia? A lesson from a recent study
Source: Biomedica. 2019 Dec 30;39(4):811–5. (PMC7363341)
Supplement: Related texts

# REPLY

**Authors:** Ingrid Johana Roldán, Rodrigo Castaño, María Cristina Navas

**Affiliations:** 1 Grupo de Gastrohepatología, Facultad de Medicina, Universidad de Antioquia, Medellín, Colombia Universidad de Antioquia Grupo de Gastrohepatología Facultad de Medicina Universidad de Antioquia Medellín Colombia

Medellín, October 31st., 2019

Dear Dr. Pellicano,

We would like to thank you for your interest and comments about our manuscript, which we found extremely useful ^8^. Since Colombia is a country with a high gastric cancer incidence we agree with your observation: “The *Helicobacter Pylori* eradication could lower the morbidity rate for this cancer. However, this rate diminution has been shown to have a dramatic downward trend because of the antibiotic resistance” ^9^. Therefore, we must implement optimal eradication regimens according to the pertinent antibiotics.

Worldwide (with rare exceptions) the use of clarithromycin regimens are no longer appropriate because of the low eradication rates (<80%) ^10^^,^^11^. Even more concerning, the alternative levofloxacin therapies (quadruple, sequential, concomitant and triple therapies) efficacy has decreased ^12^^,^^13^. While the resistance increases, the standard triple therapy success has decreased below 60% ^9^^,^^14^^-^^18^, The *H. pylori* empirical treatment has contributed to the misuse of antibiotics. Although the latest international consensus reports suggest selection of treatment according to local resistance patterns ^19^^-^^27^*H. pylori* sensitivity test is rarely performed.

The standard triple therapy is no longer use in Europe, and the quadruple therapy is now used instead ^19^. Nevertheless, since there is not consensus with this treatment a random combination is used worldwide, especially in regions with high resistance rates leading to the increase of quinolones and rifabutin resistance ^21^.

Unfortunately, in our experience most of the patients with failure treatment have shown multiple concurrent clarithromycin and metronidazole resistance, or even a triple resistance to clarithromycin, metronidazole, and fluoroquinolone (according to the culture susceptibility test) ^14^. As a result of antibiotics misuse, concomitant quadruple therapy is rapidly losing its efficacy.

Since 2015, Kioto´s *H. pylori* consensus defined it as an infectious disease regardless of the symptoms and complications ^29^. Maastricht´s V Consensus recommended that after the second-line treatment failure, *H. pylori* treatment should be guided according to the sensitivity tests ^19^. If the correct antibiotics are chosen for the first therapy the success rate is higher, while after first therapy failure the bacteria will probably develop antibiotic resistance and it would be harder to eliminate.

Bismuth is a medicament very effective to treat *H. pylori* infection and most of the consensuses recommend it because resistance against it has not been described yet ^19^^,^^8^^,^^26^^,^^27^. However, since bismuth is not absorbed it is not effective against intracellular and pericellular bacteria. Therefore, bismuth should be used in combination with additional medicaments to successfully eradicate the infection. Dore, *et al.,* demonstrated that adding bismuth to the triple therapy could increase the curation rate against resistant strains, but bismuth is rarely added to the first-line *H. pylori* triple treatment ^30^.

In our practice, as second-line therapy and increasingly more frequent as first-line therapy, the proton pump inhibitors (PPI), amoxicillin, levofloxacin and bismuth combined treatment achieve over 90% success rates in clinical trials performed since 2010 ^31^. We are currently working on a project trying this therapy with our patients because *H. pylori* will not become resistant to bismuth and it could prevent *Clostridium difficile* complications.

Besides choosing the correct antibiotics according to the sensitivity tests, the acid gastric inhibition plays a key role in treatment success. Therefore, choosing the correct PPI with the higher effectiveness over the acid and a lower influence for the host´s CYP2C19 polymorphisms (rabeprazole and esomeprazole) could improve the cure rate ^28^. Maastricht´s V Consensus establishes that a high PPI dose controls better the gastric pH, thus increasing the therapy efficacy ^19^. Since the acid inhibition power differs in different PPI, duplicating the standard dose of any of these PPI would provide a better outcome ^32^.

As a summary, *H. pylori* is an infectious disease and its eradication can be achieved (≥95% cure rate) with well-designed therapies based not only in antibiotic selection (precise selection) according to the antimicrobial sensitivity tests and cultures, but also with the patient´s adherence to the treatment and the correct PPI dose. Due to the high resistance, gastroenterologists must handle it as an infectious disease and change the empirical treatment model for a precision therapy guided by antimicrobial susceptibility tests. The treatment program needs to be applied in the local, regional and national environment to track the *H. Pylori* resistance patterns to antibiotics.

 Sincerely yours,

## References

[8] Roldán IJ, Castaño R, Navas MC (2019). Mutaciones del gen ARN ribosómico 23S de Helicobacter pylori asociadas con resistencia a claritromicina en pacientes atendidos en una unidad de endoscopia de Medellín, Colombia. Biomédica.

[9] Savoldi A, Carrara E, Graham DY, Conti M, Tacconelli E (2018). Prevalence of antibiotic resistance in Helicobacter pylori: A systematic review and meta-analysis in World Health Organization Regions. Gastroenterology.

[10] Argueta EA, Moss SF (2019). Treatment of Helicobacter pylori. Curr Opin Gastroenterol.

[11] O’Connor A, Liou JM, Gisbert JP, O’Morain C (2019). Treatment of Helicobacter pylori infection 2019. Helicobacter.

[12] Boyanova L, Hadzhiyski P, Kandilarov N, Markovska R, Mitov I (2019). Multidrug resistance in Helicobacter pylori: Current state and future directions. Expert Rev Clin Pharmacol.

[13] Fernández-Salazar L, Valle-Muñoz J (2019). Treating Helicobacter pylori infection in the face of growing antibiotic resistance. Revista Española de Enfermedades Digestivas.

[14] Arévalo A, Otero WA, Trespalacios AA (2019). Helicobacter pylori: resistencia múltiple en pacientes de Bogotá, Colombia. Biomédica.

[15] Alba C, Blanco A, Alarcón T (2017). Antibiotic resistance in Helicobacter pylori. Curr Opin Infect Dis.

[16] Smith SM, O’Morain C, McNamara D (2019). Helicobacter pylori resistance to current therapies. Curr Opin Gastroenterol.

[17] Fiorini G, Zullo A, Saracino IM, Pavoni M, Vaira D (2018). Antibiotic resistance pattern of Helicobacter pylori strains isolated in Italy during 2010-2016. Scand J Gastroenterol.

[18] Seo JW, Park JY, Shin TS, Kim JG (2019). The analysis of virulence factors and antibiotic resistance between Helicobacter pylori strains isolated from gastric antrum and body. BMC Gastroenterol.

[19] Malfertheiner P, Megraud F, O’Morain CA, Gisbert JP, Kuipers EJ, Axon AT (2017). Management of Helicobacter pylori infection-the Maastricht V/Florence Consensus Report. Gut.

[20] Otero WA, Trespalacios AA, Otero L, Vallejo MT, Torres-Amaya M, Pardo R (2015). Guía de práctica clínica para el diagnóstico y tratamiento de la infección por Helicobacter pylori en adultos. Rev Col Gastroenterol.

[21] Kato M, Ota H, Okuda M, Kikuchi S, Satoh K, Shimoyama T (2019). Guidelines for the management of Helicobacter pylori infection in Japan: 2016. Helicobacter.

[22] Chey WD, Leontiadis GI, Howden CW, Moss SF (2017). ACG Clinical Guideline: Treatment of Helicobacter pylori infection. J Gastroenterol.

[23] Bosques-Padilla FJ, Remes-Troche JM, González-Huezo MS, Pérez-Pérez G, TorresLópez J, Abdo-Francis JM (2018). The fourth Mexican consensus on Helicobacter pylori. Rev Gastroenterol Mex.

[24] Liu WZ, Xie Y, Lu H, Cheng H, Zeng ZR, Zhou LY (2018). Fifth Chinese National Consensus Report on the management of Helicobacter pylori infection. Helicobacter.

[25] El-Serag HB, Kao JY, Kanwal F, Gilger M, LoVecchio F, Moss SF (2018). Houston Consensus Conference on testing for Helicobacter pylori infection in the United States. Clin Gastroenterol Hepatol.

[26] Fallone CA, Chiba N, van Zanten SV, Fischbach L, Gisbert JP, Hunt RH (2016). The Toronto Consensus for the treatment of Helicobacter pylori infection in adults. Gastroenterology.

[27] Gisbert JP, Molina-Infante J, Amador J, Bermejo F, Bujanda L, Calvet X (2016). IV Spanish Consensus Conference on Helicobacter pylori infection treatment. Gastroenterol Hepatol.

[28] Pellicano R, Zagari RM, Zhang S, Saracco GM, Moss SF (2018). Pharmacological considerations and step-by-step proposal for the treatment of Helicobacter pylori infection in the year 2018. Minerva Gastroenterol Dietol.

[29] Sugano K, Tack J, Kuipers EJ, Graham DY, El-Omar EM, Miura S (2015). Kyoto global consensus report on Helicobacter pylori gastritis. Gut.

[30] Dore MP, Lu H, Graham DY (2016). Role of bismuth in improving Helicobacter pylori eradication with triple therapy. Gut.

[31] Gisbert JP, Romano M, Gravina AG, Solís-Muñoz P, Bermejo F, Molina-Infante J (2015). Helicobacter pylori second-line rescue therapy with levofloxacin- and bismuth-containing quadruple therapy, after failure of standard triple or non-bismuth quadruple treatments. Aliment Pharmacol Ther.

[32] Graham DY, Lu H, Dore MP (2019). Relative potency of proton-pump inhibitors, Helicobacter pylori therapy cure rates, and meaning of double-dose PPI. Helicobacter.
